# Pulmonary Exposure to Magnéli Phase Titanium Suboxides Results in Significant Macrophage Abnormalities and Decreased Lung Function

**DOI:** 10.3389/fimmu.2019.02714

**Published:** 2019-11-28

**Authors:** Dylan K. McDaniel, Veronica M. Ringel-Scaia, Holly A. Morrison, Sheryl Coutermarsh-Ott, McAlister Council-Troche, Jonathan W. Angle, Justin B. Perry, Grace Davis, Weinan Leng, Valerie Minarchick, Yi Yang, Bo Chen, Sky W. Reece, David A. Brown, Thomas E. Cecere, Jared M. Brown, Kymberly M. Gowdy, Michael F. Hochella, Irving C. Allen

**Affiliations:** ^1^Department of Biomedical Sciences and Pathobiology, VA-MD College of Veterinary Medicine, Virginia Tech, Blacksburg, VA, United States; ^2^Graduate Program in Translational Biology, Medicine and Health, Virginia Tech, Blacksburg, VA, United States; ^3^Analytical Research Laboratory, VA-MD College of Veterinary Medicine, Virginia Tech, Blacksburg, VA, United States; ^4^Department of Materials Science and Engineering, Virginia Tech, Blacksburg, VA, United States; ^5^Department of Human Nutrition, Foods, and Exercise, Virginia Tech, Blacksburg, VA, United States; ^6^National Center for Earth and Environmental Nanotechnology Infrastructure, Virginia Tech, Blacksburg, VA, United States; ^7^Department of Pharmaceutical Sciences, University of Colorado, Anschutz Medical, Aurora, CO, United States; ^8^Key Laboratory of Geographic Information Science of the Ministry of Education, School of Geographic Sciences, East China Normal University, Shanghai, China; ^9^Department of Applied Physical Sciences, University of North Carolina at Chapel Hill, Chapel Hill, NC, United States; ^10^Department of Pharmacology & Toxicology, Brody School of Medicine, East Carolina University, Greenville, NC, United States; ^11^Department of Geosciences, Virginia Tech, Blacksburg, VA, United States

**Keywords:** cytotoxicity, air pollution, nanoparticle, Ti_*x*_O_2*x*−1_, *in vivo*, environmental exposure

## Abstract

Coal is one of the most abundant and economic sources for global energy production. However, the burning of coal is widely recognized as a significant contributor to atmospheric particulate matter linked to deleterious respiratory impacts. Recently, we have discovered that burning coal generates large quantities of otherwise rare Magnéli phase titanium suboxides from TiO_2_ minerals naturally present in coal. These nanoscale Magnéli phases are biologically active without photostimulation and toxic to airway epithelial cells *in vitro* and to zebrafish *in vivo*. Here, we sought to determine the clinical and physiological impact of pulmonary exposure to Magnéli phases using mice as mammalian model organisms. Mice were exposed to the most frequently found Magnéli phases, Ti_6_O_11_, at 100 parts per million (ppm) via intratracheal administration. Local and systemic titanium concentrations, lung pathology, and changes in airway mechanics were assessed. Additional mechanistic studies were conducted with primary bone marrow derived macrophages. Our results indicate that macrophages are the cell type most impacted by exposure to these nanoscale particles. Following phagocytosis, macrophages fail to properly eliminate Magnéli phases, resulting in increased oxidative stress, mitochondrial dysfunction, and ultimately apoptosis. In the lungs, these nanoparticles become concentrated in macrophages, resulting in a feedback loop of reactive oxygen species production, cell death, and the initiation of gene expression profiles consistent with lung injury within 6 weeks of exposure. Chronic exposure and accumulation of Magnéli phases ultimately results in significantly reduced lung function impacting airway resistance, compliance, and elastance. Together, these studies demonstrate that Magnéli phases are toxic in the mammalian airway and are likely a significant nanoscale environmental pollutant, especially in geographic regions where coal combustion is a major contributor to atmospheric particulate matter.

## Introduction

Coal combustion is a significant component of the global energy portfolio and accounts for approximately 30% of the world's overall energy needs (1). The global use of coal has continued to increase over the last half century, especially in developing countries, such as China and India, due in part to abundant supplies and favorable economic factors (1). The burning of coal on such a massive global scale has also resulted in a range of detrimental environmental and human health concerns. While the long-term effects of increased coal burning, such as rising greenhouse gas emissions and climate change are clearly a concern, the much more visible short-term consequences associated with air pollution must also be addressed. Coal-burning is a significant contributor to atmospheric particulate matter with aerodynamic diameter <2.5 μM (PM_2.5_), which is the most concerning fraction for human health (2).

The smallest fraction of PM_2.5_ is ultrafine nanoscale PM associated with air pollution. These nanoparticles have highly significant biological impacts and can have detrimental effects on human health (3). These mostly incidental nanomaterials are unintentionally produced as byproducts of anthropogenic processes and typically enter the environment shortly after generation (4). Until recently, the majority of incidental nanomaterials either went unnoticed or were not evaluated for potential detrimental impacts on environmental health. However, we now know that many of these nanomaterials can contribute disproportionately to environmental chemistry impacting multiple earth systems (4). Likewise, due to their nano-scale size, these particles can readily penetrate many of the physiological and cellular barriers used to protect biological systems. For example, depending on size, shape, and composition, many nanoscale particles can readily enter the airway and gastrointestinal tract of mammals, translocate epithelial cell barriers, access the bloodstream, and affect the function of vital body systems (3). The convergence of nanoscience, environmental health, and biomedical research are beginning to provide significant insight into the impacts of incidental nanomaterials across earth systems and in human health (4).

As evidence of the progress being made in identifying and characterizing new incidental nanomaterials, recent work identified novel titanium suboxides, defined as Magnéli phases (Ti_*x*_O_2*x*−1_), which are generated during coal combustion (1). Ti_*x*_O_2*x*−1_ is produced during coal combustion at specific temperatures and oxygen fugasities from stoichiometric TiO_2_ titanium oxide that is a common accessory mineral in nearly all coals worldwide (1). Magnéli phases were originally discovered during the investigation of a coal ash spill into the Dan River (North Carolina, USA), where these novel titanium suboxides were found downstream from the spill site (5). Magnéli phases were observed in the size ranges of a few tens to hundreds of nanometers and exhibit a unique electron diffraction pattern compared to anatase and rutile, the two most common titanium oxide minerals found in coal (5). Since the discovery of Magnéli phases in this riverine environment, subsequent analyses of coal ash samples from power plants throughout the United States and China, each utilizing various types and compositions of coal, revealed that Magnéli phases are widespread and were present at every site tested (5). Magnéli phases were found with compositional ranges between Ti_4_O_7_ and Ti_9_O_17_, with the most frequent being Ti_6_O_11_ (5). Subsequent analyses of power plant stack emissions, sludge from waste water treatment plants, and road dust from Chinese urban areas were all found to contain Magnéli phases (5).

Together, these data illustrate that Magnéli phases generated as incidental nanoparticles through the combustion of coal are widespread in the environment. However, there is currently a paucity of data related to the *in vivo* relevance and physiological effects of Magnéli phases in the mammalian respiratory system. In the current manuscript, we demonstrate that Magnéli phases are concentrated and ultimately sequestered in lung associated macrophages. Magnéli phase phagocytosis significantly impairs mitochondrial function and stimulates reactive oxygen species (ROS) production by the macrophages. Ultimately, these trigger pathways associated with apoptosis and lung injury. Consistent with these findings, mice chronically exposed to Magnéli phases demonstrate significantly decreased lung function. Together, these data reveal the significant impact of these incidental nanoparticles on overall respiratory function and provide further evidence of the need for improved environmental monitoring to screen for these and similar materials.

## Materials and Methods

### Magnéli Phase Fabrication and Characterization

Magnéli phases were synthesized using a tube furnace (diameter = 8.9 cm) with a heating and cooling rate of 5°C min^−1^ and an N_2_ atmosphere (flow rate = 0.28 m^3^ min^−1^) as previously described (1). Heating and cooling processes were isothermal at the target temperature for 2 hours (h). The process includes heating pulverized coal with TiO_2_ nanoparticles. Magnéli phases were produced using commercial P25 nanoparticles, which is a mixture of the 80% anatase and 20% rutile forms of TiO_2_. Magnéli phase samples were characterized using a scanning transmission electron microscope operating at 200 kV and equipped with a silicon drift detector-based Energy Dispersive X-ray Spectroscopy (EDS) system as previously described (1).

### Experimental Animals

All mouse studies were approved by the respective Institutional Animal Care and Use Committees (IACUC) at Virginia Tech and East Carolina University, and were conducted in accordance with the *Federal NIH Guide for the Care and Use of Laboratory Animals*. All studies utilized age and gender matched wild type C57Bl/6J mice.

### Cytokine and Lactate Acid Dehydrogenase (LDH) Assessments in Bone Marrow-Derived Macrophages (BMDMs)

BMDMs were harvested from mice using standard protocols (6). Briefly, harvested bone marrow cells were cultured for 6 days in Dulbecco's Modified Eagle Media (DMEM) supplemented with 10% fetal bovine serum (FBS), L-Glutamine, 1 × penicillin/streptomycin, and 20% L929-conditioned medium. After this 6-day incubation, BMDMs were re-plated at 250,000 cells/well and were allowed to adhere to the plate for 24 h. Cells were then treated with different concentrations of Magnéli phase particles (0, 1, 10, 100, or 1,000 ppm) overnight. Following these treatments, cell-free supernatant was collected, and cytokine levels were assessed via enzyme-linked immunosorbent assay (ELISA). Lipopolysaccharide (LPS) was used at 1 μg/mL as a positive control for cytokine release. LDH activity was measured in the supernatant to assess cytotoxicity.

### Transmission Electron Microscopy to Assess Magnéli Phase Phagocytosis

Following treatment with Magnéli phases, treatment media was discarded and BMDMs (1 × 10^6^ cells/well) were fixed with 2.5% Gluteraldehyde in 0.1 M Sodium Cacodylate directly in the cell culture plate for at least 1 h. Following fixation in 2.5% glutaraldehyde in 0.1 M Na cacodylate, samples were washed two times in 0.1 M Na cacodylate for 15 min each, post-fixed in 1% OsO_4_ in 0.1 M Na cacodylate for 1 h and washed two times in 0.1 M Na cacodylate for 10 min each. Fixative was removed and cells were dehydrated with increasing concentrations of ethanol for 15 min each as follows (15, 30, 50, 70, 95, and 100%). The dehydration was completed with propylene oxide for 15 min. The samples were then infiltrated with a 50:50 solution of propylene oxide:Poly/Bed 812 (Polysciences Inc., Warrington, Pennsylvania, USA) for 6–24 h followed by complete infiltration with a 100% mixture of Poly/Bed 812 for 6–12 h. The samples were embedded in fresh 100% Poly/Bed 812 using Beem embedding capsules (Ted Pella Inc., Redding, California, USA) overnight. These were then placed into a 60°C oven for at least 48 h to cure and harden. The embedded samples were trimmed and thin (60–90 nm) sections were cut and collected on copper grids (Electron Miscroscopy Sciences). These sections were stained with aqueous uranyl acetate and lead citrate and examined and photographed using a JOEL JEM 1400 transmission electron microscope.

### Mitochondrial Stress Test

Oxygen Consumption Rate (OCR) of BMDMs after exposure to nanoparticles was determined using a Seahorse XF96 Extracellular Flux Analyzer (Agilent Technologies). Seahorse 96-well cell culture microplates were seeded at 100,000 cells per well and subjected to a 24-h incubation with Magnéli phases. Following the incubation, cells were washed with OCR assay media (1 mM pyruvate, 2 mM glutamine, and 10 mM glucose) twice and then immersed in a total volume of 180 μL of media in each well immediately before the assay. Plates were then placed in the XF96 to establish a basal level of respiration. Afterwards, three separate injections were performed: the ATPase inhibitor oligomycin (1 μM), the mitochondrial uncoupler carbonilcyanide p-triflouromethoxyphenylhydrazone (FCCP) (3 μM) and the mitochondrial electron transport chain complex III inhibitor antimycin A (2 μM), respectively. Each condition was measured three times before moving to the next injection. Data is represented as pmol of oxygen per minute.

### Mitochondrial Membrane Potential and ROS Assessments

Cells were grown following the conditions described above and plated at 100,000 cells per well in black walled, clear, flat bottom Corning Costar 96-well microplates. Images were collected in three different channels with a 60 × objective using Hoechst 33258 (100 nM) to counterstain nuclei, dichlorofluorescein (DCF) (100 μM) to monitor reaction to H_2_O_2_ injury, and tetramethylrhodamine, methyl ester (TMRM) (10 nM) to assess mitochondrial membrane potential. The following excitation/emission filters were selected to collect the fluorescence signal for each channel on the instrument; DAPI (Excitation 390.0 nm/18.0 nm bandpass, Emission 435.0/48.0 nm bandpass), FITC_511 (Excitation 475.0 nm/28.0 nm bandpass, 511.0 nm/23.0 nm bandpass), Cy3 (Excitation 542.0 nm/27.0 bandpass, Emission 587.0 nm/45.0 nm bandpass), respectively. Prior to the start of the imaging protocol, wells were treated with TMRM and DCF for 30 min, protected from light, in a 37°C/5% CO_2_ incubator and washed twice with phosphate buffered saline (PBS). Sequential images were taken in 10 fields of view for each channel in each well containing cells. After a basal level of fluorescence was obtained for each field, all wells were treated with 500 μM H_2_O_2_ to induce injury and a second round of images were obtained following the same procedure. Images were analyzed in an automated fashion using GE's InCarta software version 1.6. Multiple parameters were collected from each plate by creating custom “masks” that incorporated the fluorescent target of interest. Data are expressed as the mean pixel value under the mask minus mean pixel value for the local background or, more simply, Intensity-Background.

### Quantification of Titanium Concentration in Tissues Using Inductively Coupled Plasma Mass Spectrometry (ICP-MS)

ICP-MS analysis was performed on Agilent 7900 ICP-MS operating in helium collision mode to reduce/remove polyatomic interferences. Data was acquired in spectrum mode and utilized a 3-point peak pattern for quantitative analysis. Each data point acquisition had three replicates and utilized 250 sweeps per replicate. Titanium levels were monitored at both 47 and 49 m/z to additionally check for potential interferences at either value. The instrumental parameters for ICP-MS are shown in Supplemental Figure S2A.

Scandium (45 m/z) was used as an on-line addition internal standard. A calibration curve that ranged from 0.04 to 10 ppb titanium in solution was used to quantify titanium in the samples according to their response ratio with the scandium internal standard (Ti/Sc). An Anton-Parr Multiwave GO microwave system that utilized modified Teflon (PTFE-TFM) microwave vessels was used in the microwave digestions. This microwave oven provides feedback from the digestion conditions based on temperatures and whether excessive venting was detected.

Ultrapure water was purified with a Millipore MilliQ water purification system set at 18.2 MOhm. Single element stock solutions of 1,000 ppm titanium (Ti) and scandium (Sc) for ICP-MS analysis were obtained from Inorganic Ventures. Nitric acid (67–70 w/w%) and hydrofluoric acid (47–51 w/w%) were both trace metals grade and obtained from Fisher Scientific. Suprapur hydrogen peroxide (30 w/w%) was also trace metals grade and obtained from Merck. Lung tissue samples were stored in a freezer at −20°C until analysis. When ready, the samples were removed from the freezer and allowed to come to room temperature. The entirety of the provided samples was weighed directly into PTFE-TFM microwave vessels and ranged from approximately 200–300 mg.

One milliliter of ultrapure water (18.2 MΩ), 1.5 mL of 70% (w/w) nitric acid (HNO_3_), 0.3 mL of 10% (w/w) hydrofluoric acid (HF), and 0.2 mL of 30% w/w hydrogen peroxide (H_2_O_2_) were added to each vessel. The vessels were then capped appropriately and loaded into the carousel of the microwave digestion system. The samples were digested using a temperature gradient that ramped from room temperature to 180°C over 20 min, then held at 180°C for 10 min before cooling back to 60°C over 10 min. All samples digested clear and colorless, with no visible particulates in solution.

Each of the digested samples was decanted into fresh, separate 15 mL polypropylene (PP) tubes, and their corresponding digestion vessels were rinsed with 2 mL of 2% (w/v) HNO_3_ and decanted into their respective tubes for a 5 mL total volume. The concentrated samples were then vortexed and shaken to mix immediately before being diluted 1:100 in 2% (w/v) HNO_3_ to ensure homogeneity of the dilute samples. The 1:100 dilution was necessary to reduce the total concentration of HF within the samples themselves so that it did not attack the quartz portions of the ICP-MS instrumentation.

### Intratracheal (i.t.) Administration and Imaging of Magnéli Phases in the Lung

Wild-type C57Bl/6 mice were treated with one dose of 100 ppm of Magnéli phases using i.t. administration, following anaesthetization with isoflurane. Mice were euthanized using CO_2_ and lungs were harvested 7 days following i.t. administration. To assess chronic effects of Magnéli phases in the lung, mice were treated i.t. with 100 ppm of Magnéli phases three times per week. Lungs were harvested at 6 weeks after the initial treatment. Hematoxylin and eosin (H&E) stained lung slides were evaluated using darkfield microscopy (Cytoviva, Auburn, AL, USA) and images were collected at a magnification of 100X. Magnéli phase localization and lung histopathology were also evaluated by a board-certified veterinary pathologist using H&E stained sections prepared from formalin fixed, paraffin embedded tissues. Immunohistochemistry was conducted on the formalin fixed, paraffin embedded tissues using an APAF1 polyclonal antibody (Invitrogen; PA5-85121) and rabbit specific HRP/DAB (ABC) Detection IHC kit (Abcam).

### Gene Expression Profiling and Pathway Analysis

Total RNA was harvested from whole lungs using FastRNA Pro Green Kit following manufacturer's protocols (MP Biomedicals). Total RNA was pooled from 3-8 individual mice per group for RT^2^ Profiler PCR Array Platform (QIAgen) cDNA reaction. Gene expression was evaluated using PAMM-212Z and PAMM-052Z arrays (Qiagen) following manufacturer's protocols. The gene list for these arrays and functional categories are available through the manufacturer (Qiagen). Ingenuity Pathways Analysis (IPA) and the manufacturer's array software (Qiagen) was used to analyze gene expression data and conduct pathway analysis. IPA data were ranked and evaluated based on *z*-score as previously described by the research team (7–9).

### Pulmonary Function Testing

Male C57Bl/6J mice were exposed to PBS or Magnéli phases three times/week for 6 weeks (18 total doses). Briefly, mice were anesthetized with isoflurane and received Magnéli phases (100 ppm) in 50 μl by i.t. administration as described above for the chronic exposure assessments. Pulmonary function testing was performed 24 h following the last exposure. All mice were anesthetized with tribromoethanol (TBE) (400 mg/kg), tracheostomized, and baseline pulmonary function was recorded using the FlexiVent system (SCIREQ, Montreal, QC, Canada) (10, 11).

Mice were ventilated with a tide volume of 10 ml/kg at a frequency of 150 breaths/min and a positive end expiratory pressure (PEEP) of 3 cm H_2_O to prevent alveolar collapse. In addition, mice were paralyzed with pancuronium bromide (0.8 mg/kg) to prevent spontaneous breathing. Electrocardiogram (EKG) was monitored for all mice to determine anesthetic depth and potential complications that could have arisen during testing. A deep inflation perturbation was used to maximally inflate the lungs to a standard pressure of 30 cm H_2_O followed by a breath hold of a few seconds to establish a consistent volume history. A snapshot perturbation maneuver consisting of a three-cycle sinusoidal wave of inspiration and expiration to measure total respiratory system resistance (R), dynamic compliance (C), and elastance (E). A Quickprime-3 perturbation, which produced a broadband frequency (0.5–19.75 Hz) over 3 s, was used to measure Newtonian resistance (Rn), which is a measure of central airway resistance. All perturbations were performed until three acceptable measurements with coefficient of determination (COD) ≥0.9 were recorded in each individual subject.

After three baseline measurements of lung function using “snapshot” and “Quickprime-3” perturbations, mice were challenged with aerosolized saline or cumulative doses of 1.5, 3, 6, 12, and 24 mg/ml methacholine (MCh) (Sigma-Aldrich, St. Louis, MO) generated by an ultrasonic nebulizer for 10 s without altering the ventilation pattern (10–12). These challenges were followed by assessments with “snapshot” and “Quickprime-3” perturbations every 30 s. Between each dose of methacholine, a deep inflation perturbation was initiated to reset lung hysteresis. For each animal, all perturbations were performed until six acceptable measurements with COD ≥0.9 were recorded.

### Replicates

All studies were repeated at least three independent times unless noted.

### Statistical Analysis

Single data point comparisons were evaluated by Student's two-tailed *t*-test. Multiple comparisons were evaluated for significance using Analysis of Variance (ANOVA) followed by either Tukeys or Newman–Keuls post-test, as appropriate. Graphs and statistical analyses were performed and generated using GraphPad Prism software 8 (GraphPad, San Diego, CA). All data are presented as mean ± the standard error of the mean (SEM) with *p* ≤ 0.05 considered significant. Data shown are representative of at least three independent studies.

## Results

### Magnéli Phases Are Cytotoxic in Murine Bone Marrow-Derived Macrophages

**Figure 1:**
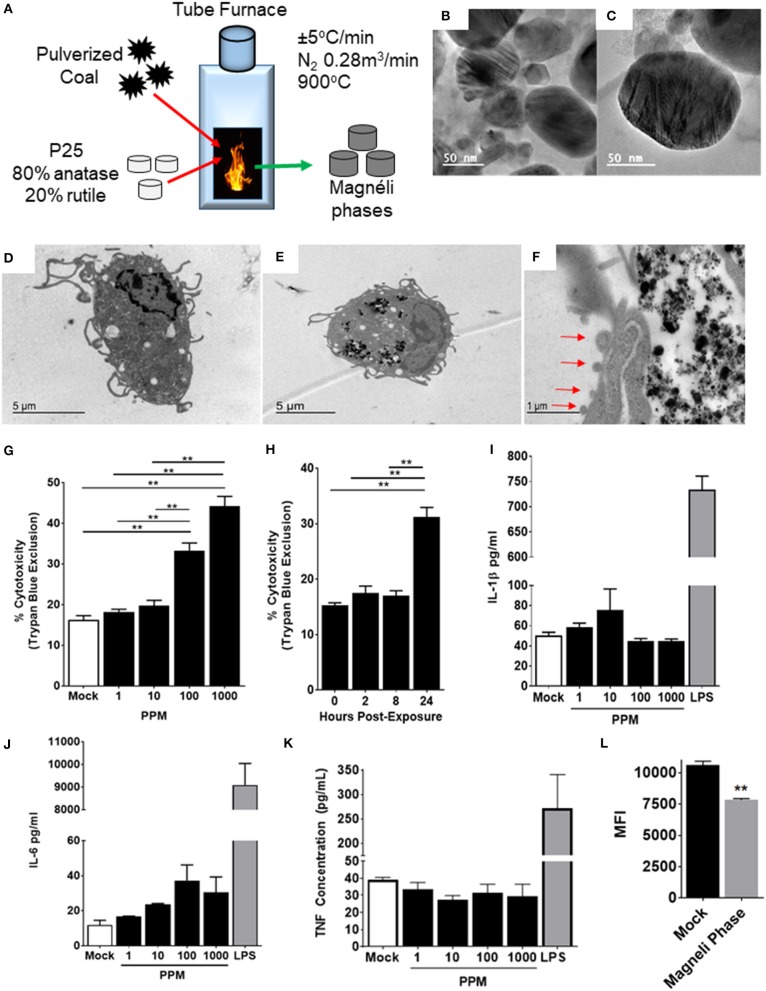
Magnéli phase phagocytosis results in increased cell death in bone marrow-derived macrophages. **(A–C)** Characterization of Magnéli phases used in this study. **(A)** Schematic illustrating Magnéli phase generation. **(B,C)** TEM images of Magnéli phases formed by annealing P25 TiO_2_ nanoparticles with coal in a pure N_2_ atmosphere for 2 h at 900°C. Electron diffraction patterns were characteristic of Magnéli phases and confirmed these as predominately Ti_6_O_11_ particles. Particles were between 10 and 200nm in size. **(D)** Un-treated bone marrow-derived macrophages (1 × 10^6^ cells/well) and **(E)** macrophages treated with Ti_6_O_11_ (1, 10, 100, or 1,000 ppm) were visualized using TEM (Scale bar: 5 μm). Magnéli phases appear as punctate dark dots in the macrophages and are concentrated in phagolysosomes. **(F)** Macrophages containing Magnéli phases demonstrate morphological features consistent with apoptosis, including cell shrinking and membrane blebbing (red arrows) (Scale bar: 1μm). **(G,H)** Cytotoxicity was evaluated using trypan blue exclusion **(G)** across the Ti_6_O_11_ dose range and **(H)** at 100 ppm over a 24 h time course. **(I,J)** Inflammation was evaluated by assessing the production of pro-inflammatory cytokines, such as **(I)** IL-1β, **(J)** IL-6, and **(K)** TNF in the cell free supernatant following exposure to different doses of Magnéli phases. **(L)** Macrophage function was evaluated by assessing the ability of macrophages to phagocytose fluorescent *Escherichia coli* 24 h post-exposure to 100 ppm Magnéli phases. Data are expressed as mean ± SEM (*n* = 3 independent experiments). **p < 0.01.

In initial toxicity studies, Magnéli phases (Ti_6_O_11_) were found to be toxic to dechorionated zebrafish embryos at 100 ppm (1). Thus, we chose this formulation and dose as the focus of our subsequent studies. Utilizing previously described methods, we generated Magnéli phases that were predominately composed of Ti_6_O_11_ (1) (Figure 1A). The resultant nanoparticles were confirmed by electron microscopy analysis and X-ray diffraction patterns, as previously described (1) (Figures 1B,C). These nanoparticles have excellent light absorption in the near-infrared, UV, and visible light range (1). Likewise, the Magnéli phases also display a low amount of photocatalytic activity, as previously reported (1). The resultant nanoparticles used in our studies ranged in size from a few tens of nm to hundreds of nm (1).

Once inhaled, nanoparticles are rapidly phagocytosed by resident macrophages in the lungs, which represent the predominate cell type responsible for neutralization and clearance (13). To evaluate the effect of Magnéli phases on these cells, we generated primary bone marrow derived macrophages (BMDMs) using established protocols (13). The BMDMs were treated with varying doses of Ti_6_O_11_ Magnéli phases across a 0–1,000 ppm range and over a 24-h time course (Figures 1D–H). Transmission electron microscopy (TEM) assessments revealed Magnéli phases, concentrated in phagolysosomes within treated macrophages at all concentrations and timepoints evaluated (Figures 1D,E). Morphology assessments did not appear to show high levels of macrophage activation following phagocytosis. However, at 100 ppm, a significant number of macrophages demonstrated morphology consistent with increased cell death, specifically apoptosis (Figure 1F). Several features were noted, including cell shrinkage, loss of membrane integrity, and membrane blebbing (Figure 1F). To quantify cell death, we utilized trypan blue exclusion and manual counting using a hemacytometer. Here, we observed a dose dependent increase in cytotoxicity between 10 and 1,000 ppm, ranging from 19.75 ± 2.63% to 44.25 ± 4.787%, respectively (Figure 1G). The average cell death over the same 24-h time range in the mock treated macrophages was 16.25 ± 2.062% (Figure 1G). Macrophage cell death did not initiate immediately, requiring 24 h post-exposure to peak (Figure 1H). Using 100 ppm, cell death was assessed over a time course, with almost double the number of macrophages undergoing cell death at 24 h post-exposure (15.25 ± 0.957% mock vs. 31.25 ± 3.403% Ti_6_O_11_ Magnéli phase treatment) (Figure 1G). Together, these data show that Ti_6_O_11_ Magnéli phases, at levels observed in the environment as incidental nanoparticles generated through the industrial burning of coal, are cytotoxic to mammalian macrophages.

**Figure 2:**
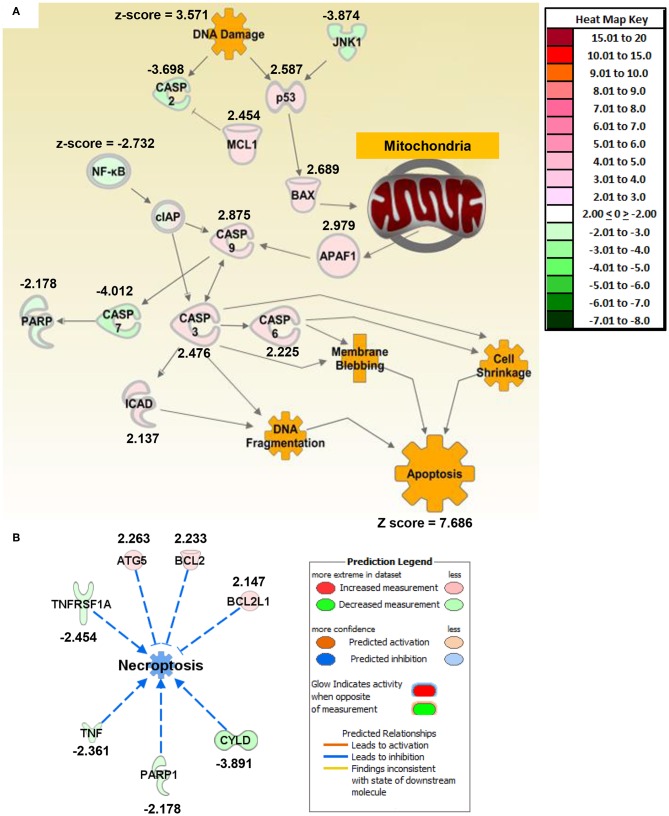
Macrophage exposure to Magnéli phases activates gene expression profiles associated with apoptosis and mitochondria dysfunction. **(A)** Gene expression was evaluated using real time PCR based Superarrays (Qiagen). For each gene target on the array, fold change was calculated based on ΔΔCt values. Genes found to be ±2-fold change in expression from untreated specimens were defined as significant. The resultant data was evaluated using Ingenuity Pathway Analysis (IPA) to define pathways and global correlations between gene expression profiles and biological functions. Cell death signaling, specifically apoptosis, was the top pathway up-regulated in the macrophages following Magnéli phase exposure. IPA further identified gene expression profiles consistent with mitochondria dysfunction as a potential factor associated with the increased apoptosis signaling. **(B)** Pathways associated with inflammation and specifically inflammation associated cell death (i.e., pyroptosis and necroptosis) were significantly down-regulated at the level of gene transcription. Either fold change in gene expression or z-score values are displayed for each node as appropriate.

Nanoparticle exposure typically drives macrophage activation and the production of inflammatory mediators (13). The cells generated using the methods detailed here are skewed toward M1 polarized, pro-inflammatory macrophages (6). Likewise, the cell death induced by the Magnéli phases were originally predicted to generate damage associated molecular patterns, such as aberrant ATP production or Calcium efflux similar to that described for A549 cells (14), that should drive innate immune system signaling in the macrophages. Here, we monitored the generation of several potent pro-inflammatory mediators, including IL-1β, IL-6, and TNF (Figures 1I–K). Consistent with electron microscopy findings indicating apoptosis as the method of cell death and the lack of morphological features associated with macrophage activation, we did not observe any statistically significant increases in either gene transcription by rtPCR or at the protein level by ELISA for any pro-inflammatory mediators evaluated (Figures 1I–K). Indeed, at the gene transcription level, we observed several mediators and transcription factors actively down-regulated (Figure 2). These data indicate that cell death and inflammation induced by Magnéli phases are not linked. Despite higher levels of cytotoxicity, cell death and Magnéli phase exposure are not sufficient to significantly activate elements of the innate immune system associated with inflammation.

Previous studies have shown that nanoparticle phagocytosis by macrophages can impair their function, resulting in greater susceptibility to a myriad of pathogens (15, 16). To evaluate macrophage functionality 24 h following either mock or Magnéli phase exposure, live cells containing nanoparticles were sorted and co-exposed to fluorescently labeled *E. coli*. In macrophages lacking the Magnéli phases, we observed a significant increase in mean fluorescence intensity (MFI: 10,630 ± 290) compared to those containing the nanoparticles (7,826 ± 114.2) (Figure 1L). Thus, cells containing Magnéli phases were significantly less efficient at bacterial phagocytosis, implying that nanoparticle sequestration has a deleterious impact on macrophage function.

### Magnéli Phase Phagocytosis Activates Apoptosis Signaling Pathways Modulated Through the Mitochondria

To better define mechanisms underlying Magnéli phase cytotoxicity, we profiled gene expression and utilized Ingenuity Pathway Analysis to decipher changes in gene networks and pathways associated with a range of biological functions in macrophages. BMDMs were collected 24 h post-exposure to 100 ppm Ti_6_O_11_. Total RNA was collected and gene expression was profiled using rtPCR based gene expression arrays (Qiagen). Changes in gene expression were defined as significant if the change was ±2-fold different between the mock and treated cells. Based on the pattern of gene expression changes, apoptosis signaling was the top canonical pathway that was significantly increased following Magnéli phase exposure (Figure 2; Supplemental Figure S1A). Individually, there were no clear networks or relationships identified between the top 10 dysregulated genes (Supplemental Figure S1B). However, analysis of the congregate gene expression patterns and networks revealed a strong relationship between genes associated with apoptosis signaling with a focus on mitochondria (Figure 2A). Here, we observed significant increases in gene expression associated with both pro-apoptotic BCL-2 family members, specifically *Bax* (Figure 2A). Up-stream analysis revealed a significant increase in *p53* signaling and down-stream analysis revealed a significant increase in *Apaf1* (Figure 2A). Consistent with these findings, Caspases-3,−6, and−9 were all found up-regulated following Magnéli phase exposure, while Caspase-7 was significantly downregulated (Figure 2A). We also observed a significant up-regulation in the gene encoding ICAD, which is associated with DNA fragmentation during apoptosis (Figure 2A). In addition to apoptosis, pathway analysis further revealed that inflammation and inflammatory forms of cell death, such as necroptosis, were significantly down-regulated following Magnéli phase exposure (Figures 2A,B). While we realize the general limitations of utilizing gene expression data to define cell death pathways, the data indicates significant changes in genes associated with apoptosis pathways and is consistent with the electron microscopy and inflammation data (Figure 1). Beyond cell death, very few additional pathways were found to be significantly impacted following Magnéli phase phagocytosis at the time points and conditions evaluated here, with a notable exception being a general downregulation of genes associated with NF-κB signaling (Figure 2A). This finding is consistent with the lack of inflammation and cytokine responses observed following exposure (Figures 1I,J). To better evaluate our findings compared to those previously reported for TiO_2_ exposure, we compared our findings to gene expression patterns associated for TiO_2_ in the IPA database (Supplemental Figure S1C). In general, many of the same pathways are altered for both Magnéli phases and TiO_2_, including the down-regulation of *Tnf* and the up-regulation of *Ifng* (Supplemental Figure S1C). However, the major difference between Magnéli phases and TiO_2_ is associated with Caspase-3/-7 regulation. Caspase-7 is down regulated in both cases; but, Caspase-3 is significantly up-regulated following Magnéli phase exposure and is central in the apoptosis pathways identified (Figure 2A; Supplemental Figure S1C). This is in contrast to the down-regulation of Caspase-3 and cell death mechanisms previously described following TiO_2_ exposure (17). Together, these data suggest that macrophage cell death induced by Magnéli phase exposure occurs though the mitochondrial pathway of apoptosis and is associated with the activation of pro-apoptotic BCL-2 family members, including a potential p53-BAX-APAF1 axis.

### Magnéli Phase Exposure Results in Significant Mitochondrial Dysfunction

**Figure 3:**
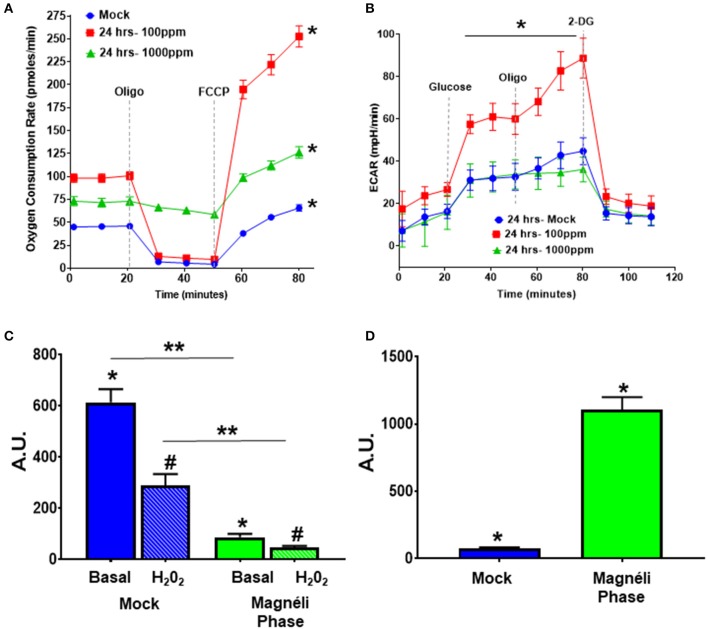
Alterations in cellular energetics and mitochondrial membrane potential in macrophages treated with Ti_6_O_11_. **(A)** Oxygen consumption rate (OCR) and **(B)** extracellular acidification rate (ECAR) were measured using Agilent Seahorse XF96 Analyzer in mock and Ti_6_O_11_ treated BMDMs at either 100 ppm or 1,000 ppm. **(C)** Mitochondrial membrane potential was evaluated using tetramethylrhodamine (TMRM) in macrophages treated with vehicle (blue) or 100 ppm Magnéli phases (green), with H_2_O_2_ used to induce maximum loss of membrane potential in each group. **(D)** Cellular oxidative stress was evaluated using dihydrodichlorofluorescein (DCF) fluorescence. All data are expressed as mean ± SEM (*n* = 3/group). AU, Arbitrary Units. *p < 0.05, #*p* < 0.05, **p < 0.01.

Previous studies have revealed that nanoparticles can significantly impair mitochondria function and the convergence of signaling pathways on the mitochondria identified in the gene expression profiling and pathway analysis together warrant higher resolution functional assessments. We first utilized an Agilent Seahorse XF cell mitochondrial stress test to evaluate the oxygen consumption rate (OCR) in BMDMs treated for 24 h with either mock, 100 ppm, or 1,000 ppm Magnéli phases (Figure 3A). At both doses, the basal respiration and the maximal respiratory capacity (MRC) were significantly increased in BMDMs treated with Magnéli phases compared to mock (Figure 3A). Cells treated with 1,000 ppm of Magnéli phases did not show the typical decline in OCR after the addition of oligomycin (Oligo), a known ATP synthase inhibitor, indicating an increase in proton leak in the mitochondria (Figure 3A). Proton leak is typically associated with mitochondrial damage and stress (18, 19). Likewise, these cells only showed a minimal change in MRC following the addition of the proton ionophore FCCP (Figure 3A), which likely reflects the significant increase in cells undergoing cell death. The BMDMs treated with 100 ppm Magnéli phases showed the most dramatic shifts, with the highest level of basal respiration and the largest MRC increase, following FCCP treatment (Figure 3A). The impressive increase in MRC suggests that Magnéli phase exposure resulted in a substantially higher reserve capacity in BMDMs, which is also likely associated with the increased energy demands of the cells following increased damage, stress, and cell death. Complementing the OCR data, we also evaluated the extracellular acidification rate (ECAR), which is an assessment of glycolysis. While we did not observe significant differences in ECAR following exposure to Magnéli phases at 1,000 ppm, we did observe a significant increase following exposure to 100 ppm (Figure 3B). These data indicate a shift in the substrate utilization, likely due to increased cellular metabolism in cells treated with 100 ppm Magnéli phases. Together, these data indicate that Magnéli phase exposure results in altered mitochondrial bioenergetics that can significantly contribute to damage associated processes, such as reactive oxygen species (ROS) generation and the activation of cell death cascades.

Because proton leak is also a sign of mitochondrial damage and stress, we next investigated mitochondrial membrane potential (MMP), as decreases in MMP are associated with mitochondrial membrane damage and changes in mitochondrial function. The cell-permeant dye, tetramethylrhodamine (TMRM) accumulates in active mitochondria with healthy MMPs. A decrease in TMRM fluorescence indicates a loss of mitochondrial membrane potential. BMDMs treated with 100 ppm Magnéli phases had significantly lower TMRM fluorescence (86.26 ± 13.06 AU) compared to mock (613.60 ± 51.89 AU) (Figure 3C), indicating treatment significantly altered mitochondrial membrane potential. The subsequent addition of H_2_O_2_ to each sample was used as a positive control and resulted in significant decreased TMRM fluorescence compared to basal fluorescence in the mock treated cells, with a much smaller decrease in the cells treated with Magnéli phases (Figure 3C). Together, these data suggest that Magnéli phase exposure results in a significant decrease in mitochondrial membrane potential, and confirms severe mitochondrial membrane damage.

The production and modulation of ROS is a common biological response in macrophages to nanoparticle exposure, is associated with cytotoxicity, and is commonly involved in mitochondrial stress (20–22). Increased ROS production is a significant driver of cell death and stress following TiO_2_ exposure (20). A previous study assessed ROS levels in A549 alveolar epithelial cell lines following TiO_2_ and undefined Magnéli phase exposure (14). Under the conditions evaluated, the TiO_x_ nanoparticles increased intracellular Ca^2+^ that was associated with low levels of cytotoxicity, but no significant changes on ROS production were observed (14). Due to the inherent differences in nanoparticle responses between different cell types and the potent cytotoxic effects of dysregulated ROS production, we next evaluated this response following exposure to Ti_6_O_11_ nanoparticles in macrophages. For this we utilized another cell-permeant dye, 2′,7′-dichlorofluorescin diacetate (DCFDA), which is oxidized by ROS into the fluorescent DCF. BMDMs challenged with 100 ppm Magnéli phases for 24 h had a significantly higher level of ROS production (1,105 ± 94.17) compared to mock exposed cells (76.39 ± 7.22) (Figure 3D). Taken together, these data suggest that macrophages treated with Magnéli phases show increased ROS production and loss of mitochondrial membrane potential after 24 h. All of which are indications that these particles induce mitochondrial stress and are consistent with the induction of mitochondria-mediated apoptosis following Magnéli phase exposure.

### Magnéli Phases Are Sequestered in Lung-Associated Macrophages and Concentrate in the Tissue After a Single Exposure

**Figure 4:**
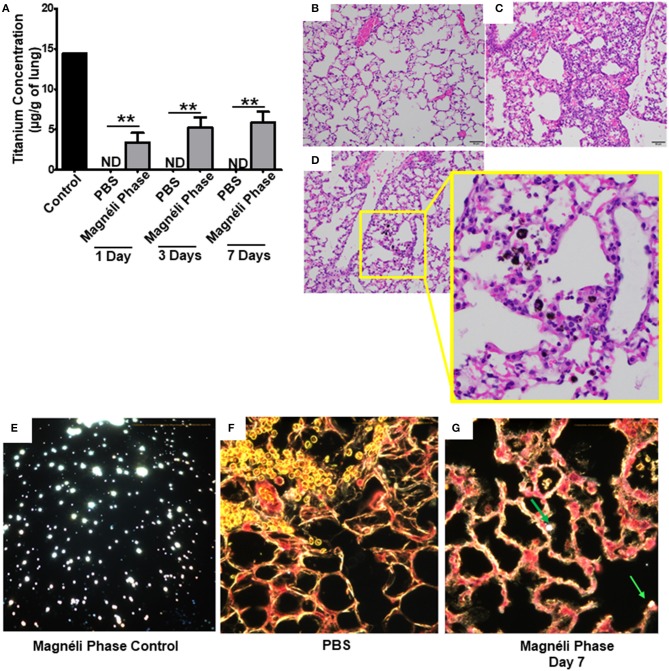
Magnéli phases concentrate in pulmonary macrophages following a single exposure and are retained in the lung. **(A)** Following a single airway exposure to 100 ppm, titanium associated with the Magnéli phases were retained in the lungs and detected using ICP-MS for over 7 days post-expsure. Control tissues were collected from animals exposed to 100 ppm Ti_6_O_11_ and immediately euthanized. **(B–D)** Representative H&E stained tissue sections used for histopathology evaluation. **(B)** Vehicle control showing no nanoparticles or pathology. **(C)** Significant airway inflammation was observed in LPS treated animals, but no evidence of inflammation was found in the mice treated with Magnéli phases at any timepoint evaluated. **(D)** Larges areas of macrophages containing Magnéli phases were found in all treated animals (yellow box). **(E–G)** The majority of pulmonary macrophages contain Magnéli phases, with no other cell types appearing to contain or associate with the nanoparticles determined using dark field microscopy. Darkfield images were taken of **(E)** Ti_6_O_11_ nanoparticles alone or the lungs of mice 7 days post-exposure to either **(F)** PBS or **(G)** 100 ppm Magnéli phases. **(G)** Particles in the tissues were identified by bright punctate dots, almost exclusively localized in macrophages. Representative H&E stained lung sections (Scale bar = 50 μm). All data are expressed as mean ± SEM (*n* = 7/group). **p < 0.01.

In the only *in vivo* toxicity study conducted to date, Magnéli phases were shown to be bioactive and toxic to zebrafish at 100 ppm (1). These studies provided the first indication that these incidental nanoparticles could be biologically active and potentially toxic to a variety of animal species, including humans. However, the impact of Magnéli phases in mammals and the mechanism of toxicity were not explored (1). Thus, we next sought to extend these findings using mice and a respiratory exposure model. Mice were exposed to 100 ppm Ti_6_O_11_ Magnéli phases in 1 × PBS via intra-tracheal administration using methods previously described to evaluate nanoparticle toxicity and trafficking in the respiratory tract (13, 23). To quantify the concentration of Magnéli phases in the lungs over time, we utilized inductively coupled plasma mass spectrometry analysis to detect titanium levels (Figure 4A; Supplemental Figure S2A). Immediately following i.t. administration, tissues including the lungs, liver, spleen, and kidneys were removed, digested, and processed for ICP-MS. These data represent the maximum deposition control (Figure 4). As expected, no titanium was detected in tissues outside of the lungs or in any tissues that were not exposed to the Magnéli phases. However, in exposed lungs, we observed 14.46 ± 4.32 μg/g of lung tissue immediately after deposition (Figure 4A). Within 24 h post-exposure, titanium levels detected in the lungs dropped to 3.40 ± 1.19 μg/g of lung tissue (Figure 4A). However, surprisingly, after this single exposure to the Ti_6_O_11_ Magnéli phases, the levels of titanium in the lungs were maintained over the course of a week with levels holding steady at 5.30 ± 1.23 μg/g (Day 3) and 5.91 ± 1.30 μg/g (Day 7) (Figure 4A). Titanium levels were below the level of detection in all of the other tissues evaluated at the time points assessed (Supplemental Figure S2B). Together, these data emphasize the potential consequences of even a single exposure to Magnéli phases, which are retained in the lungs at significant levels for an extended duration following inhalation.

We next sought to identify where in the lungs the Magnéli phases were being sequestered. We initially utilized histopathology assessments of H&E stained lung sections to evaluate both nanoparticle deposition and pathology. Lungs were fixed by gravity inflation with formalin and paraffin embedded for staining as previously described (13, 23). Lung pathology was evaluated by a board-certified veterinary pathologist (S.C.O.). Control mice received either vehicle or were challenged with a 1 mg/ml dose of LPS (Figures 4B,C). As anticipated, lung histopathology was normal in the vehicle treated animals (Figure 4B). Conversely, we observed significant airway inflammation in the LPS treated animals, characterized by predominately macrophage and neutrophil infiltration concentrated around the airways and vasculature (Figure 4C). In the experimental animal groups, we did not observe significant evidence of inflammation in the mice exposed to Magnéli phases at 1, 3, or 7 days post-exposure (Figure 4D). However, Magnéli phases were readily observed as punctate foci within cells in the lungs under light microscopy conditions (Figure 4D). Further assessments at higher magnification revealed that the vast majority of Magnéli phases were sequestered in macrophages, with no nanoparticles observed under light microscopy conditions associated with or within any other cell type in the lung or observed in the vehicle control specimens (Figures 4B,D). To confirm the association of Magnéli phases with the macrophages in the lungs, we conducted enhanced darkfield microscopy (Cytoviva) (Figures 4E–G). A reference image of Magnéli phases was generated and used to identify the nanoparticles *in vivo* (Figure 4E). As anticipated, we did not detect any Magnéli phases in the vehicle control specimens (PBS) (Figure 4F). However, consistent with our pathology assessments, Magnéli phases were confirmed in macrophages throughout the lungs and were readily identified by their distinctive punctate staining (Figure 4G; green arrow). Together, these data show that Magnéli phases are effectively phagocytosed by lung-associated macrophages; however, the nanoparticles are not effectively cleared from the tissue, remaining at steady levels over the course of at least 7 days following a single exposure.

**Figure 5:**
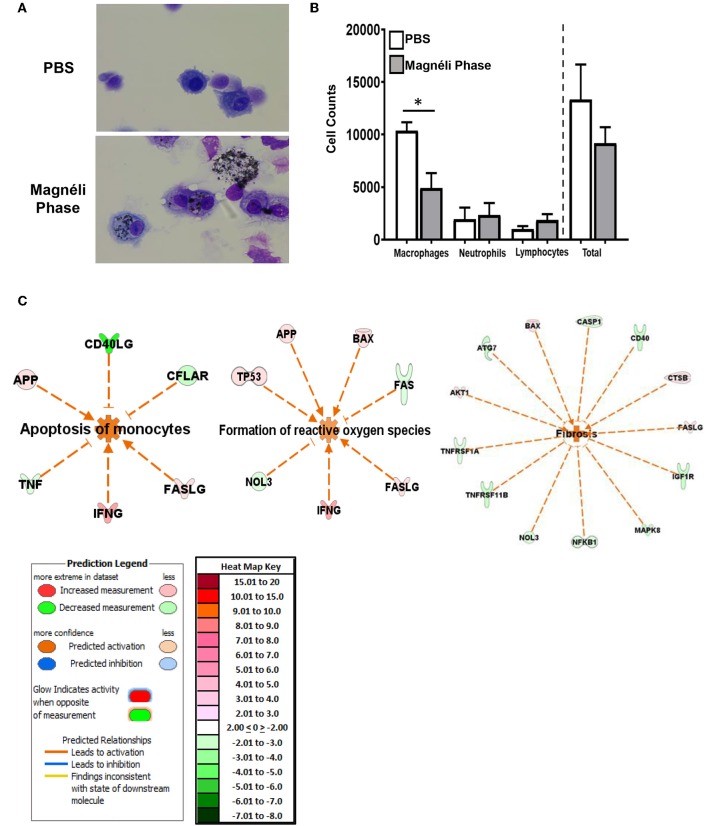
Magnéli phases concentrate in pulmonary macrophages, resulting in significant dysfunction. **(A)** Representative images of BALF cytology from PBS and Ti_6_O_11_ (100 ppm) treated mice. **(B)** Differential cell counts in the BALF after treatment with PBS, Ti_6_O_11_, or LPS. **(C)** Gene expression profiling and analysis using Ingenuity Pathway Analysis software identified gene transcription patterns associated with increases in apoptosis, formation of reactive oxygen species, and wound healing/fibrosis. Data are expressed as mean ± SEM (*n* = 3 mice per PBS treated group, *n* = 7 mice per Ti_6_O_11_ treated group, and *n* = 4 per LPS treated group). *p < 0.05.

Based on our *ex vivo* findings that Magnéli phases are cytotoxic, we next evaluated the *in vivo* impact of nanoparticle sequestration on macrophages and lung tissue. Bronchoalveolar lavage fluid (BALF) was collected following euthanasia and cellularity was evaluated following differential staining. In both the PBS and Magnéli phase treated specimens, macrophages were the dominate population of cells recovered (Figure 5A). This is in contrast to the highly inflammatory conditions observed following LPS treatment (data not shown) or following exposure to nanoparticles with immunostimulatory properties, where neutrophils are the dominate cell type observed (24–27). Magnéli phases were readily identified in macrophages collected from the lungs of exposed mice at all time points evaluated, with 71 ± 10.16% of the macrophages containing at least 1 readily observable nanoparticle under 40 × light microscopy (Figure 5A). Consistent with our *in vitro* cytotoxicity data, many of the macrophages recovered from the airways demonstrated morphological features consistent with cellular stress and death (Figure 5A). The cell populations recovered in the BALF were further quantified using a hemocytometer (Figure 5B). Macrophages, neutrophils, and lymphocytes were recovered in the BALF from mice exposed to either PBS or Magnéli phases (Figure 5B). The quantity and populations of cells recovered in BALF are routinely utilized as surrogates to characterize lung inflammation (28). Compared to findings commonly reported following exposure to immunostimulatory agents, such as LPS, and consistent with the pathology evaluations shown in Figures 4B–D, the number and populations of cells recovered following exposure to Magnéli phases indicate that these nanoparticles do not induce significant lung inflammation (Figure 5B). Indeed, assessments of inflammatory mediator levels, such as IL-6, in the BALF following either Magnéli phase exposure or LPS exposure, further confirm these findings (Supplemental Figure S3A). Consistent with the increased cytotoxicity, we observed a significant decrease in the number of macrophages recovered in the BALF from mice exposed to Magnéli phases (Figure 5B). No other cell populations appeared to be impacted (Figure 5B). Together, these data indicate that airway exposure to a single dose of Magnéli phases at a range previously found in environmental samples following incidental nanoparticle release and cytotoxic to zebrafish embryos indeed facilitates macrophage cell death in the mammalian lung. Consistent with the apoptosis findings (shown in Figures 1, 2), neither *in vivo* macrophage cell death nor exposure to the Magnéli phases result in significant inflammation.

To better define the mechanisms underlying the *in vivo* response to Magnéli phase exposure, lung tissue was collected 7 days post-exposure. Total RNA was isolated and processed for gene expression profiling using the SuperArray platform and IPA, as previously described (7–9). The gene expression profiling analysis revealed significant changes in several pathways associated with cell death and lung function. The three top pathways predicted by IPA analysis to be significantly activated 7 days post-exposure to the Magnéli phases were (1) apoptosis—specifically apoptosis of monocytes; (2) formation of ROS; and (3) fibrosis. In all three cases the combination of genes up-regulated and down-regulated were predicted to have an activating effect on the pathways identified (Figure 5C). Also, of note, no pathways were predicted to be significantly inhibited based on the gene expression patterns evaluated and no pathways associated with inflammation were significantly modulated following Magnéli phase exposure. The predicted activation of apoptosis of monocytes and formation of ROS *in vivo* are highly consistent with the findings from our *in vitro* studies and further emphasize the detrimental impact that the Magnéli phases are having on the macrophage populations in the lungs following exposure. The fibrosis gene expression signature is intriguing. The 7-day timeframe of the exposure model is far too soon for lung fibrosis to present in the mice and no pathologic features associated with fibrosis were detected in our specimens by histopathology, collagen, or fibronectin assessments (Figure 4). It is possible that we are identifying gene signatures associated with fibrosis at a very early time in the lungs, prior to pathological disease onset. However, this gene expression pattern would also be consistent with increased regeneration and repair processes in the lungs, perhaps associated with the increased cell death.

### Repeated Exposure to Magnéli Phases Results in Significant Nanoparticle Accumulation in the Lungs

**Figure 6:**
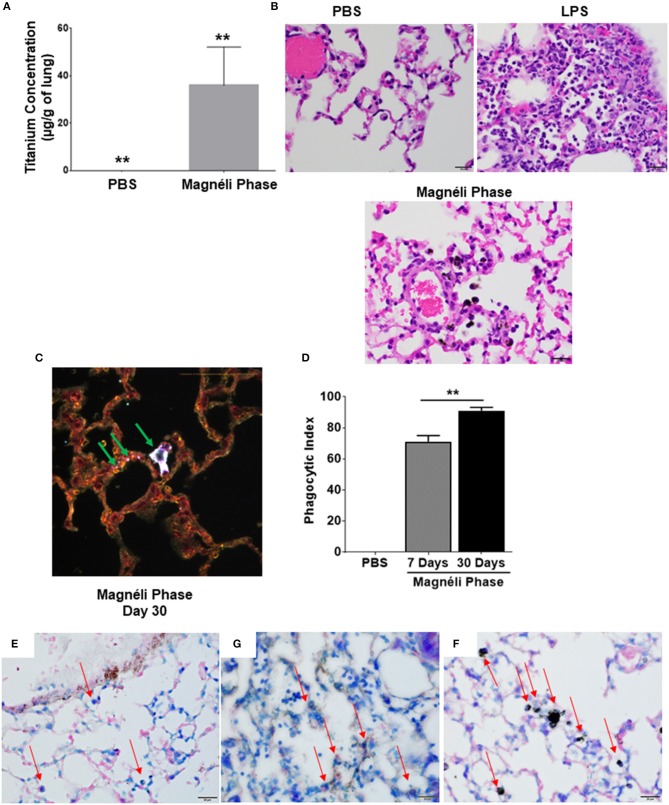
Repeated exposures concentrates Magnéli phases in pulmonary macrophages. **(A)** Using ICP-MS, we quantified the amount of titanium in the lungs following multiple airway exposures to 100 ppm Ti_6_O_11_ over a 30 day period. **(B)** Histopathology using H&E stained tissue sections reveled significant airway inflammation LPS treated animals, but no evidence of inflammation in the mice treated with Magnéli phases. Larges areas of macrophages containing Magnéli phases were found in all treated animals (Scale bar = 20 μm). **(C)** Darkfield images were taken of mouse lungs post-repeated exposures, on day 30. Particles in the tissues were identified by bright punctate dots. Almost all particles were concentrated in macrophages (green arrows). However, small numbers of Ti_6_O_11_ nanoparticles were found associated with alveolar epithelial cells (green arrows). Representative H&E stained lung sections (Scale bar = 50 μm). **(D)** Phagocytic Index (number of BALF macrophages containing Magnéli phases per 100 cells). **(E–G)** APAF1 immunohistochemistry staining from **(E)** saline, **(F)** LPS, and **(G)** Magnéli Phase exposed lungs. **(G)** Following Magnéli Phase exposure, only endothelial cells and macrophages containing particles were broadly positive for APAF1. **(E)** In saline exposed mice, only endothelial cells were positive for APAF1; **(F)** whereas, LPS exposure resulted in a range of cell types staining positive for APAF1. Macrophages in each image are identified by red arrows. All data are expressed as mean ± SEM (*n* = 7/group). **p < 0.01.

In most cases of incidental nanoparticle release into the environment, biological exposure to Magnéli phases are expected to occur repeatedly and for an extended duration. Thus, we next sought to determine the impact of chronic airway exposure to the Ti_6_O_11_ nanoparticles over a 30-day time period. Under these conditions, we observed a significant increase in titanium concentrations in the lungs, which accumulated to levels that were ~6-fold higher (35.82 μg/g of lung) than levels observed after a single exposure (5.92 μg/g of lung) (Figure 6A). Similar to the findings from the single exposures, pathology assessments of the lungs again revealed that the majority of the Magnéli phases detectable under bright-field microscopy were sequestered in lung-associated macrophages (Figure 6B). Despite the increased concentrations of nanoparticles in the lungs, we did not observe any pathologic features associated with increased inflammation or differences in pro-inflammatory mediators, such as IL-6, IL-1β, or TNF levels, in the lungs (Figure 6B; Supplemental Figures S3A–F). Pathology assessments revealed no evidence of interactions with other cell types beyond the macrophages under bright-field microscopy. To better define nanoparticle-cell interactions, we evaluated specimens using enhanced dark field microscopy (Cytoviva) (Figure 6C). Consistent with our bright field data and similar to what we observed following the single exposure, the Magnéli phases are overwhelmingly concentrated in macrophages in the lung (Figure 6C). It should be noted that we did identify punctate staining associated with alveolar epithelial cells, indicating that under chronic exposure conditions, there are some interactions between other cell types in the lungs and the Ti_6_O_11_ nanoparticles (Figure 6C). However, compared to the macrophages, Magnéli phase interactions with these cells were more sporadic and involved far fewer nanoparticles per cell (Figure 6C). Using cells recovered following BALF and evaluated following differential staining assessments, the number of macrophages containing Magnéli phases per 100 cells was determined and used to generate a phagocytic index (29). Following the single exposure to Magnéli phases, we calculated the phagocytic index to be 71.14 ± 3.84 on day 7, with the majority of macrophages recovered containing >1 nanoparticle per cell (Figure 6D). However, following the multiple exposures, the phagocytic index significantly increased to 91.00 ± 2.17 on day 30, again with almost all macrophages recovered containing >1 nanoparticle per cell (Figure 6D). Together, these data are consistent with the Magnéli phases being sequestered in the macrophages. Following cell death, the nanoparticles likely continue to concentrate in the lungs as cells undergoing apoptosis and associated debris (including the Magnéli phases) are further phagocytosed by additional macrophages, creating a positive feedback loop.

Our gene expression profiling data indicated that this sequestration resulted in apoptosis. To further evaluate this, we conducted immunohistochemistry targeting APAF1, which is an important mediator in mitochondria mediated apoptosis and was significantly up-regulated at the gene expression level (Figure 2A). Immunohistochemistry revealed that Ti_6_O_11_ treatment resulted in significant increases in APAF1^+^ cells in the lungs. Consistent with previous data, endothelial cells have high levels of APAF1^+^ staining, which was observed in the Saline treated animals (Figure 6E). However, we did not observe any significant staining in other cell types, including macrophages (Figure 6E). In LPS treated lungs, we observed robust staining in multiple cell types in the lungs, including endothelial cells, epithelial cells, macrophages, and neutrophils (Figure 6G). However, in the Magnéli phase treated animals, we did not observe the same level of widespread staining (Figure 6F). Rather, the APAF^+^ cells were predominately limited to the endothelial cells and macrophages containing Magnéli phases (Figure 6F). Also, of note, only the macrophages containing Magnélli phases were APAF^+^. Together, these data are consistent with our other findings localizing the *in vivo* effects of the Magnélli phases to the macrophage compartment in the lungs.

### Magnéli Phase Exposure Attenuates Lung Function and Impacts Airway Pathophysiology

Based on the data above, we predicted that exposure to Magnéli phases would negatively impact mammalian lung function. To directly test this hypothesis, we determined the impact of multiple exposures of Ti_6_O_11_ nanoparticles over a 30 day period of time on basal airflow and airway mechanics utilizing a computer-controlled small-animal ventilator with highly sensitive pressure transducers (Flexivent) to quantify airway opening pressures, volume, and airflow (10, 11). In addition to basal measurements, we also determined whether exposure to these Magnéli phases modulates lung pathophysiology and airway mechanics following exposure to the bronchoconstricting agent methacholine. To evaluate airway mechanics, a snapshot perturbation maneuver that consisted of a three-cycle sinusoidal wave of inspiration and expiration was used to measure total respiratory system resistance (R), dynamic compliance (C), and elastance (E). A Quickprime-3 perturbation, which produced a broadband frequency (0.5–19.75 Hz) over 3 s, was used to measure Newtonian resistance (Rn), which is a measure of central airway resistance. For the methacholine studies, after three baseline measurements of lung function, mice were challenged with aerosolized saline or increasing doses of 1.5, 3, 6, 12, and 24 mg/ml of methacholine, generated by an ultrasonic nebulizer for 10 s without altering the ventilation pattern, followed by assessments every 30 s (30, 31). Between each dose of methacholine, a deep inflation perturbation was initiated to reset lung hysteresis.

**Figure 7:**
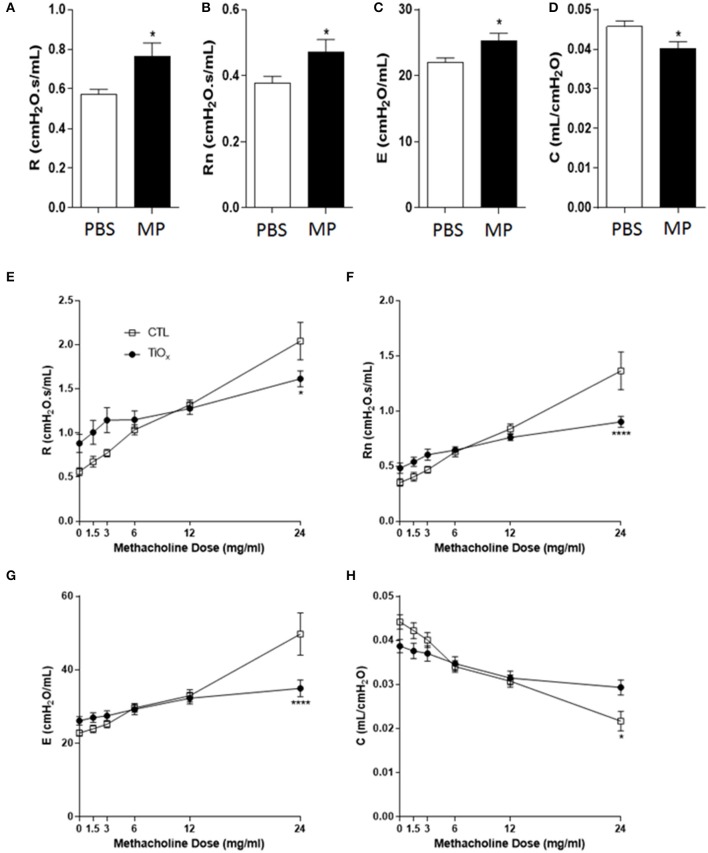
Chronic exposure to Magnéli phases significantly attenuates lung function. **(A–D)** Baseline pulmonary function parameters including **(A)** total respiratory system resistance (R), **(B)** Newtonian resistance (Rn), **(C)** elastance (E), and **(D)** dynamic compliance (C) were measured by FlexiVent in control and Ti_6_O_11_ exposed mice. **(E–H)** Assessment of AHR to methacholine (MCh) in control and Ti_6_O_11_ exposed mice. **(E)** Total respiratory system resistance (R), **(F)** Newtonian resistance (Rn), **(G)** elastance (E), and **(H)** dynamic compliance (C) dose response curves following challenge with increasing concentrations of MCh in aerosolized saline (0, 1.5, 3, 6, 12, and 24 mg/ml) for control and Ti_6_O_11_ exposed mice were examined on FlexiVent. All data are expressed as mean ± SEM (*n* = 7/group). *p < 0.05, ****p < 0.0001, compared to the PBS treated control group.

Consistent with our hypothesis, exposure to Magnéli phases resulted in significant increases in basal resistance and Newtonian resistance (Figures 7A,B). These data indicate that the accumulation of Ti_6_O_11_ nanoparticles significantly attenuates baseline lung function. The increased basal resistance and Newtonian resistance suggests that the airway diameter and lung volumes are reduced under baseline conditions. We also observed an increase in basal elastance and the converse decrease in compliance (Figures 7C,D). These findings reflect defects in alveolar and elastic tissue in the lungs and indicate that exposure to Magnéli phases results in increased lung stiffness and reduced lung expansion abilities. Beyond baseline assessments, we also sought to evaluate lung function following methacholine challenge, which is commonly used to induce smooth muscle constriction in the lungs. Despite the increased baseline resistance and Newtonian resistance, the lungs of mice exposed to Magnéli phases showed minimal increases in airway constriction following methacholine challenge (Figures 7E,F). This is in contrast to the unexposed animals, which demonstrated the anticipated dose dependent increases (Figures 7E,F). These data indicate that the lungs of these mice lack the capacity to properly function and are likely at maximum for resistance and Newtonian resistance. Methacholine challenge also results in increased elastance and decreased compliance in untreated mice (Figures 7G,H). However, similar to the resistance measures, Magnéli phase exposure resulted in minimal changes in elastance and dynamic compliance, indicating a stiffening of the lungs (Figures 7G,H). Together, these data are consistent with Magnéli phase exposure resulting in significant detrimental changes in lung mechanics and function.

## Discussion

In initial toxicity studies, Magnéli phases (Ti_6_O_11_) were found to be toxic to dechorionated zebrafish embryos at 100 ppm, without concurrent solar radiation (1). This is in contrast to TiO_2_ nanoparticles, which are photocatalytically active and toxic at the same concentrations in conditions where solar radiation is present (1). The mechanism of cytotoxicity associated with TiO_2_ has previously been associated with the increase in ROS generation (32, 33). Prior to data presented in the current study, the physiological impact in mammalian cells and cytotoxicity mechanisms associated with Magnéli phases were unclear. To date, only one additional study has explored the cytotoxicity of these nanoparticles in mammalian cells (14). The cytotoxicity of three different Magnéli phases and two different TiO_2_ formulations were evaluated utilizing A549 cells, which are a human lung alveolar epithelial cell line (14). In these studies, rather than using specific formulations of Magnéli phases, the nanoparticles were evaluated based on size, ranging from 192 to 795 nm in average diameter (14). In the A549 cells, the TiO_x_ Magnéli phases demonstrated low levels of cytotoxicity in some of the size ranges evaluated and increased intracellular Ca^2+^, but no significant changes in ROS levels were observed (14). Thus, the authors concluded that in A549 cells Magnéli phases had either similar or reduced cytotoxicity compared to TiO_2_ nanoparticles with cell death associated with different mechanisms related to Ca^2+^ flux (14).

The prior studies using zebrafish embryos also provides critical insight into general *in vivo* toxicity and immediate relevance to aquatic organism exposure (1). However, the impact of Magnéli phases on other terrestrial biological systems and the extension of these findings to human physiology is somewhat limited. Similarly, the *in vitro* studies using the A549 model provides important insight related to the interaction of Magnéli phases with a human alveolar epithelial cell line and a comparison with TiO_2_ nanoparticles (14). However, while the A549 cells are certainly relevant, when nanoparticles are internalized through the airway, several other cell types beyond the alveolar epithelial cells are arguably more critical to the host response (13). Based on data presented here, interactions between Magnéli phases and alveolar epithelial cells appear quite limited *in vivo*. Our data indicates that the vast majority of nanoparticles are sequestered in lung-associated macrophages. These cells are the primary phagocytic cell in the airway and these findings are similar to other studies evaluating nanoparticle deposition in the lungs (13, 23, 24). Likewise, our data indicates that the macrophages are not able to effectively breakdown and clear the concentrated Magnéli phases from the lungs. Even more concerning, this sequestration occurs even after a single challenge and suggests that even short term, low dose exposure to Magnéli phases may pose a potential health risk.

Once phagocytosed by macrophages, our data reveals that Ti_6_O_11_ Magnéli phases are cytotoxic and induce apoptosis, which is considered a non-inflammatory form of cell death. This finding is consistent with the lack of inflammatory signaling in the *ex vivo* primary macrophages and *in vivo* findings. However, the lack of inflammation, both *in vitro* and *in vivo*, following nanoparticle exposure was quite unexpected. As discussed above, we observed active suppression of gene expression pathways associated with necroptosis (Figure 2B), which is a proinflammatory form of cell death. While this was also unexpected, similar findings have been reported for other metal-based nanoparticles. Indeed, the ability to attenuate inflammation while inducing cell death is a favorable characteristic for engineered nanomaterials generated for biomedical and therapeutic applications. For example, zinc oxide nanoparticles induce high levels of apoptosis associated with increased ROS production and significant depletion of glutathione in human pulmonary adenocarcinoma cell lines and have well defined anti-inflammatory activities (34, 35). Thus, it is tempting to speculate a similar model applies to the Magnéli phases evaluated here. Similarly, silver nanoparticles have shown effects in neutrophils that are consistent to those described here for the Magnéli phases in macrophages (36). Silver nanoparticle exposure triggers activation and maturation in specific subsets of neutrophils, followed by increased levels of IL-8 (36). However, classical pro-inflammatory pathway activation, overall inflammation and necrotic cell death are significantly attenuated following exposure (36). The implication of these data indicate that silver nanoparticles stimulate neutrophil up-take as the cell attempts to minimize inflammation and stimulate nanoparticle clearance (36). It is possible that a similar mechanism is also occurring in macrophages, where Magnéli phase nanoparticle phagocytosis is occurring, but the cells are not able to effectively degrade or clear the nanoparticles.

Mitochondria are critical in the activation of apoptosis in mammalian cells and the Bcl-2 family is central in the regulation of these processes (37). Our data reveals that this pathway is significantly dysregulated following Magnéli phase exposure and provides insight into the mechanism underlying the increased apoptosis observed following nanoparticle exposure. These data are consistent with findings associated with a diverse range of metal-based nanoparticles. For example, gadolinium oxide (Gd_2_O_3_) nanoparticles have been shown to induce apoptosis through dysregulation of BCL2/BAX signaling and ROS production in response to DNA damage in human neuronal cells (38). Likewise, similar to the gene expression pattern observed following Magnéli phase exposure, silver nanoparticle exposure also increases caspase 3, Bax, and P53 expression, increased ROS signaling, and apoptosis in the MCF-7 human adenocarcinoma cell line (39). But, as discussed above and also similar to Magnéli phases, silver nanoparticle exposure results in an attenuated immune system response (36). Interestingly, lower doses of Magneli phases (100 ppm) increased maximal respiration, consistent with the concept of mitochondrial hormesis, whereby ROS upregulate mitochondria as a compensatory mechanism to fight cellular stressors (40). This is consistent with our observation that higher doses (1,000 ppm) were associated with functional decrements, including uncoupled respiration and a loss in respiratory reserve in macrophages. Likewise, the dramatic shifts observed in the OCR and ECAR at 100 ppm further emphasize that the mitochondria are dysfunctional and indicates that Magnéli phase exposure results in significantly altered mitochondrial bioenergetics. This likely contributes to damage associated processes, such as ROS generation and the activation of cell death cascades. Together, these data support a model whereby Magnéli phases directly impair mitochondrial function following exposure and induce cell death in macrophages through the mitochondrial-mediated intrinsic apoptosis pathway.

Despite the lack of inflammation in the lungs following Magnéli phase exposure, it is clear that the sequestration and concentration of these nanoparticles and resultant macrophage cell death significantly affects lung function. We observed significant changes in resistance, compliance, and elastance at baseline and following methacholine stimulation (Figure 7). At baseline, the increase in resistance following repeated Magnéli phase exposure reflects a narrowing of the central and conducting airways, including the trachea, bronchi, bronchioles, and terminal bronchioles. Similarly, the reduction in compliance and corresponding increase in elastance at baseline reflect the need for greater pressures to increase lung volume in animals exposed to Magnéli phases. Following stimulation with methacholine, we observed predicted changes in R, Rn, E, and C in animals unexposed to Magnéli phases, reflecting normal lung function. However, in mice exposed to the nanoparticles, we observed relatively minimal changes in lung mechanics from baseline (Figure 7). This suggests that the lungs have already reached their near maximum potential for the parameters assessed in this study. Together, these consequential alterations in airway mechanics indicate that the lungs of mice exposed to Ti_6_O_11_ Magnéli phases are significantly stiffer and more constricted compared to unexposed animals. This influences both baseline lung function and normal airway contractility following stimulation. These findings are consistent with restrictive lung disease and are similar to findings reported for silica associated models of fibrosis, which are characterized by low levels of inflammation and the production of pro-fibrotic growth factors that ultimately result in fibrotic lesions due to diminished lung clearance of the silica particles (41–44). These fibrotic lesions are directly associated with diminished macrophage function. Macrophages that concentrate the silica particles are highly effective in directly stimulating fibrogenesis associated growth factors in the lungs that can impair lung function. These findings are consistent with those presented here following exposure to Magnélli phases and we speculate that a similar mechanism underlies the observed decrease in lung function.

Due to the widespread environmental distribution of incidental Magnéli phases in the air and water associated with global coal combustion, it is critical that we have a better understanding of their biological impacts. Improved understanding of the dynamics associated with nanoparticle physicochemistry, cellular, and organismal responses following exposure, environmental transport, and global distribution patterns are all critical for providing a basis for establishing future frameworks to determine potential risks to human health and inform environmental policy decisions. Our findings here are highly cautionary, as a single exposure to Magnéli phases can result in potentially harmful long-term health effects. Unfortunately, there is currently no practical way to limit the formation of Magnéli phases and other nanoparticle formation during the coal burning process. However, in countries with strong environmental regulations, most of these nanoparticles can be captured by particle traps prior to final emission of exhaust gas. While we have provided the first evidence of the detrimental impacts of Magnéli phase exposure in mammals, it is clear that further toxicology studies are necessary and future assessments of the impact of these nanoparticles on human health is warranted.

## Data Availability Statement

The datasets generated for this study are available on request to the corresponding author.

## Ethics Statement

The animal study was reviewed and approved by Virginia Tech IACUC East Carolina University IACUC.

## Author Contributions

DM and IA designed, performed the *in vitro* and *in vivo* experiments, analyzed and interpreted the data, prepared the figures, and wrote the paper. VR-S, HM, WL, VM, YY, BC, JB, and MH were responsible for designing and executing specific studies associated with Magnéli phase synthesis and characterization, localization, and macrophage studies. MC-T and JA were responsible for the ICP-MS analysis. TC conducted the TEM analysis. JP, GD, and DB were responsible for the mitochondria studies. SR and KG conducted the lung function studies. Pathology assessments were conducted by a board certified veterinary pathologist, SC-O. All authors have read and approved the manuscript.

### Conflict of Interest

The authors declare that the research was conducted in the absence of any commercial or financial relationships that could be construed as a potential conflict of interest.

## Supplementary Material

Supplemental Figure S1 Apoptosis signaling was significantly increased in macrophages following exposure to Magnéli phases. **(A)** IPA revealed that cell death and apoptosis signaling pathways were significantly up-regulated following Ti_6_O_11_ exposure. The top molecular and cellular functions predicted to be impacted by exposure were associated with cell death, cellular function and maintenance, DNA repair, and cellular organization. **(B)** The 10 genes most impacted (the top 5 up-regulated and down-regulated) by Magnéli phase exposure in macrophages have broad and diverse biological functions. **(C)** Macrophage responses to Magnéli phases produce a gene expression pattern similar to that observed following exposure and activation following titanium dioxide. However, Magnéli phase exposure is significantly less inflammatory (lower levels of *IFNG, IGF1*, and *FASLG*), while inducing higher levels of apoptosis and increased mitochondria dysfunction. The activation of gene networks associated with Caspase 3 is a significant difference observed here for Magnéli phases, which is down-regulated following titanium dioxide exposure.

Supplemental Figure S2 Titanium was not found in tissues and organs outside of the lungs. **(A)** Table showing ICP-MS instrument parameters used for the detection of titanium in tissue. **(B)** Blood, liver, and kidneys were evaluated for titanium levels over the course of 7 days post-exposure to a single dose of Magnéli phases at 100 ppm. All levels in all tissues were below the level of detection, with the exception of a single liver sample, shown here 3 days post-exposure.

Supplemental Figure S3 Magnéli phases are non-inflammatory. **(A,B)** Pro-inflammatory cytokine levels were evaluated by gene expression using rtPCR and protein evaluations using ELISA in all *in vitro* and *in vivo* studies. However, no significant increases in the majority of potent pro-inflammatory mediators were detected. **(A–F)** IL-6, IL-1β, and TNF levels were evaluated in bronchoalveolar lavage fluid (BALF) by ELISA following either **(A–C)** a single exposure of 100 ppm; **(D–F)** chronic exposures of 100 ppm Magnéli phases; or **(A–F)** airway exposure to 1 mg/ml of lipopolysaccharide (LPS). LPS induced a robust IL-6, IL-1β, and TNF response; however, no exposures to the Magnéli phases significantly induced these proinflammatory mediators. ^*^*p* < 0.05.
