# Supplementary material for: Pulmonary Exposure to Magnéli Phase Titanium Suboxides Results in Significant Macrophage Abnormalities and Decreased Lung Function
Source: Front Immunol. 2019 Nov 28;10:2714. doi: 10.3389/fimmu.2019.02714 (PMC6892980; doi:10.3389/fimmu.2019.02714)

Supplemental Figure S1.

A.

Top Canonical Pathways	p-value
Apoptosis Signaling	26.7
Death Receptor Signaling	23.7
Induction of Apoptosis	31.1
TNFR1 Signaling	28.0
MyC Mediated Apoptosis Signaling	20.0

Top Molecular and Cellular Functions	p-value
Cell Death and Survival	82.3
Cell Morphology	58.2
Cellular Function and Maintenance	59.2
DNA Repair	36.4
Cellular Assembly and Organization	38.2

B.

Top Genes Dysregulated	Fold Change
SYCP2	17.244
DPYSL4	7.882
IFNG	7.584
INS	7.379
JPH3	7.284
GALNT5	-2.049
KCNIP1	-2.228
TNFRSF10A	-4.008
FOXI1	-7.581
CD40LG	-7.982

C.

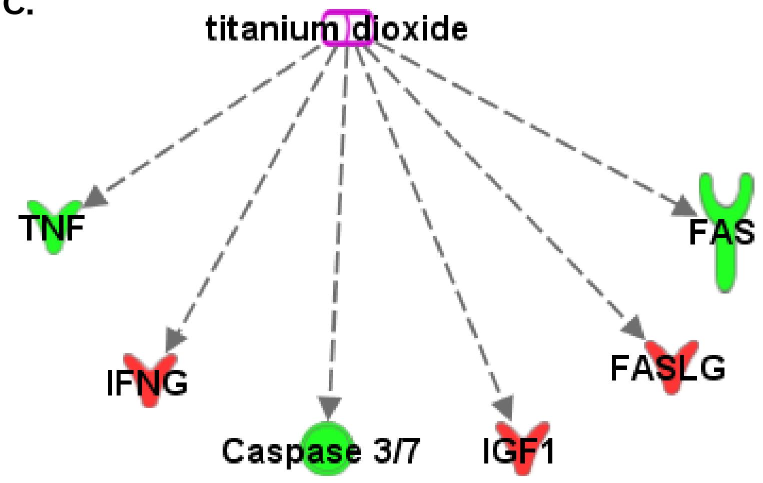

Supplemental Figure S2.

A.

Instrument Parameter	Value
RF Power	1550 W
RF Matching	1.8 V
Sampling Depth	8 mm
Carrier Gas Flow	He, 0.77 L/min
Nebulizer Pump	Peristaltic pump, 0.1 rotations/sec
Spray chamber Temp.	2 °C
Dilution Gas Flow	He, 0.26 L/min

B.

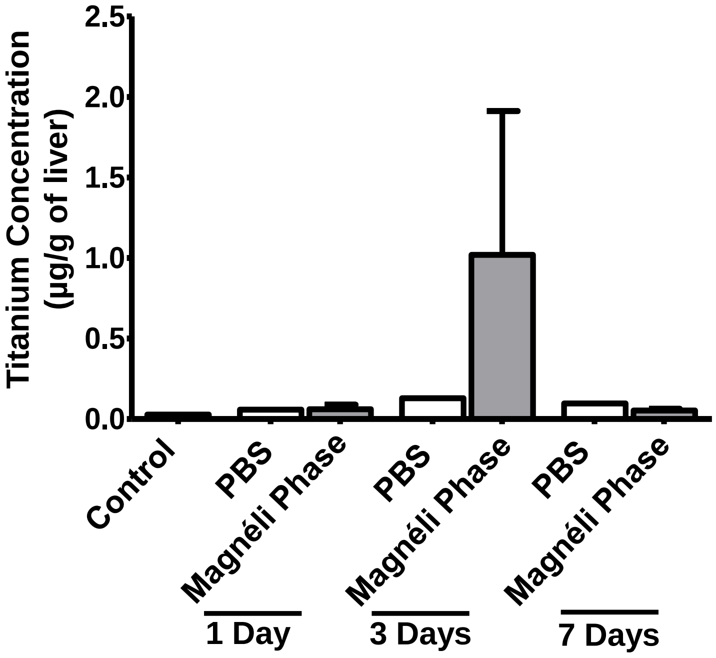

Supplemental Figure S3.

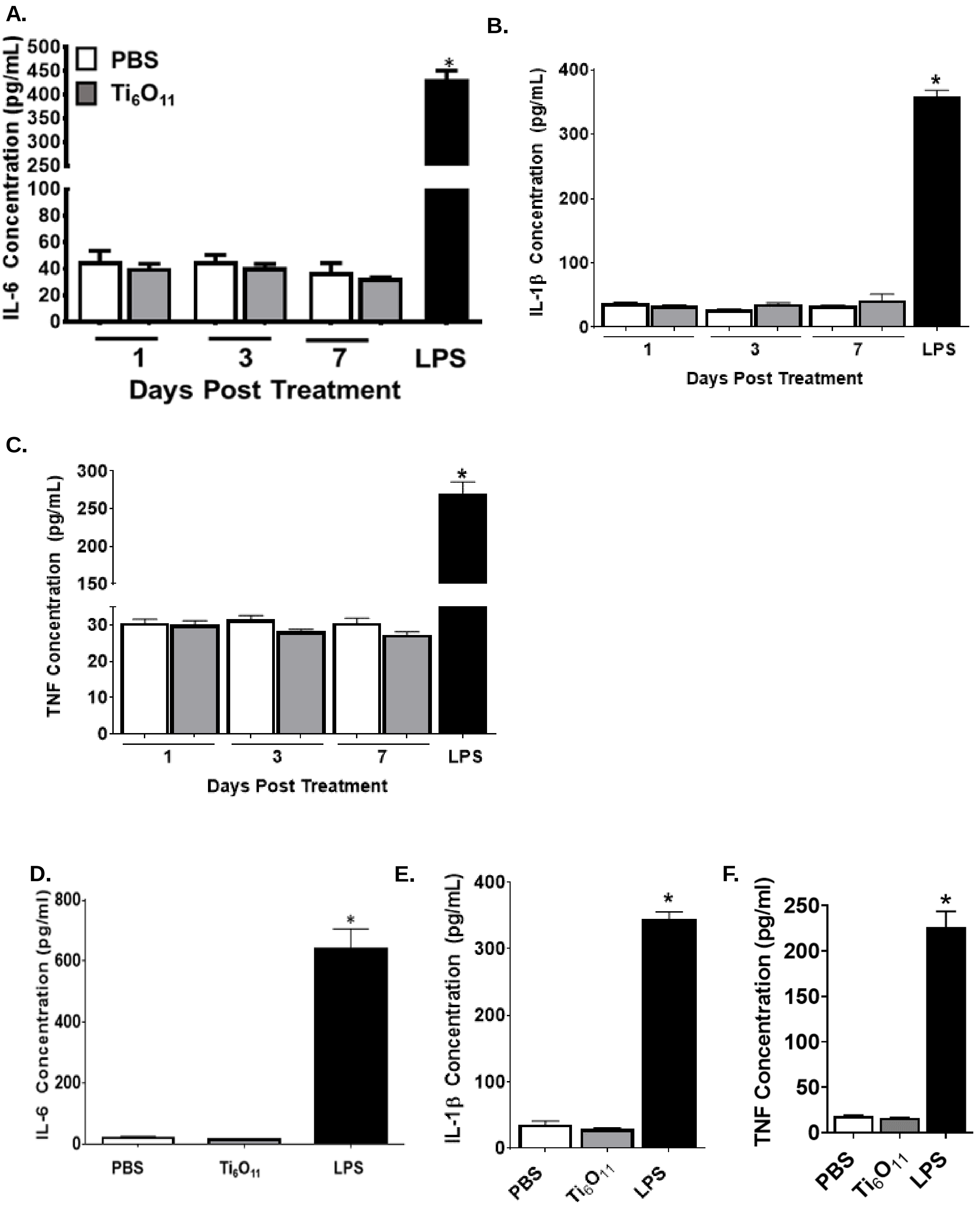

Supplement: Supplemental Figure S1 — Apoptosis signaling was significantly increased in macrophages following exposure to Magnéli phases. (A) IPA revealed that cell death and apoptosis signaling pathways were significantly up-regulated following Ti6O11 exposure. The top molecular and cellular functions predicted to be impacted by exposure were associated with cell death, cellular function and maintenance, DNA repair, and cellular organization. (B) The 10 genes most impacted (the top 5 up-regulated and down-regulated) by Magnéli phase exposure in macrophages have broad and diverse biological functions. (C) Macrophage responses to Magnéli phases produce a gene expression pattern similar to that observed following exposure and activation following titanium dioxide. However, Magnéli phase exposure is significantly less inflammatory (lower levels of IFNG, IGF1, and FASLG), while inducing higher levels of apoptosis and increased mitochondria dysfunction. The activation of gene networks associated with Caspase 3 is a significant difference observed here for Magnéli phases, which is down-regulated following titanium dioxide exposure. [file Data_Sheet_1.PDF]
